# Recent Progress in Syphilology

**Published:** 1877-10

**Authors:** 


Recent Progress in Syphilology.—The question of the unity or duality of the virus producing the different varieties of the venereal sore has been recently discussed by Mr. J. L. Milton, of London. From the consideration of a small number of cases reported by others, Mr. Milton concludes that the duality of the venereal virus is an established fact. Considering that a majority of the foremost syphilologists in England and Germany, and not a few in this country, hold opposite opinions, and probably upon equally good grounds, the question can hardly be regarded as settled.

Strong grounds in favor of the existence of a true syphilitic gonorrhœa is taken by Mr. Henry Lee, in the Hunterian Lectures delivered by him before the Royal College of Surgeons in 1875. The experiments of Prof. Tarnowsky, of St. Petersburgh, who succeeded in producing syphilitic infection by means of the blenorrhœic matter from syphilitic patients, gives considerable support to this view. Not a few American surgeons, among them Prof. Gross, hold the same opinion.

Whether induration of the initial lesion occurs prior to constitutional infection, or whether it is an indication of such infection having taken place, is considered by Auspitz. He gives a detailed account of thirty-three cases of indurated chancre, in which the initial lesion was excised at various times (four days to seven weeks) after the first appearance of the morbid process. The following results were obtained: The wound healed by first intention in six cases. In none of these eighteen cases did induration recur in the cicatrix. In the remaining fifteen cases, the cicatrix became indurated. In fourteen cases no constitutional syphilitic symptoms appeared after a sufficiently long interval of observation after the excision. In nine cases constitutional syphilis occurred despite the incision. In the remaining ten cases the result, owing to insufficient length of time for observation, and other causes, remains doubtful. From the results obtained in these cases, Auspitz concludes that the initial induration is not an expression of syphilis, but on the other hand the real *depot d'infection*, from which the organ is saturated with the virus.

The excision was usually performed by seizing the induration with a vulsellum, raising it from the surrounding healthy tissue, and cutting the button out with a pair of curved scissors. The edges of the oval wound were then united with sutures or adhesive plaster.

The hereditary transmission of syphilis has lately been investigated anew, with, as yet, no definite results. Dr. J. E. Atkinson considers the question from a histological point of view, and arrives at the conclusion that, from the biological relations of the sperm and germ-cells to epithelium and white blood-cells, syphilis may be transmitted by either parent. In an excellent report of two cases observed by himself, Dr. R. W. Taylor thinks himself justified in concluding that "syphilis may pass from father to offspring while the mother remains unaffected." The careful and complete report of these cases certainly appears to be pretty positive evidence. Kassowitz enters into by far the most extensive investigation of the subject, and concludes from his researches that syphilis may be transmitted by either parent, the other remaining unaffected. Keyfel endeavors to prove that the offspring may be infected with semen from a syphilitic father, and the mother escape the infection. Owing to the incomplete manner in which his cases are reported, however, his statistics are not of much value as testimony. Diday also tries to establish his favorite theory of infection of the mother through the fœtus; but the simple non-observance of primary lesions on the woman surely cannot be considered, in the nature of positive proof that she did not obtain her syphilis by the usual road! Oewe, after the careful examination of a number of cases occurring under his observation, concludes as follows: (*a*) Fathers suffering from latent constitutional disease have no direct part in the development of hereditary syphilis; (*b*) the children of such fathers are healthy; (*c*) hereditary syphilis invariably supposes an infected mother; and (*d*) syphilitic semen does not exert any influence on the maternal organism, either directly or indirectly (by pregnancy).

Sturgis reviews the question, taking the above mentioned views of Kassowitz for a text, and points out clearly the fallacies which the advocates of the direct paternal transmission of syphilis fall into. After a careful consideration of the whole subject, it seems to the present writer that the weight of evidence rests with those who deny the direct transmission from the father, and that satisfactory proof has not yet been furnished that a syphilitic child can be born without previous infection of the mother.

Attention has recently been called by Fournier, Rollett, and others, to the importance cf recognizing the influence of syphilis as a factor in phthisis. Fournier sums up the anatomical points of distinction between tubercle and syphiloma of the lungs as follows:

1. Situation: Tubercle involves upper part of both lungs; gummata one lung, and may be limited to a portion.

2. Number: Gummata are few, as a rule; solitary tubercle sooner or later become confluent.

3. Gummata are larger than tubercles; never miliary in form.

4. Color: Gummata are always white or yellow—never transparent like miliary tubercle.

5. Consistency: Until softening takes place, the gumma is of more equal consistence than tubercle, and when it softens does not break down wholly, owing to the capsule.

The diagnosis is difficult. Attention is called to the fact that a remarkable tolerance of the disease is sometimes seen, in which event the patient retains his flesh and strength to a considerable degree. Another point of distinction is its slow evolution, whereas it is rapid in tuberculosis. The following conclusions are offered:

1. Tertiary syphilis can produce, in the lungs, lesions which, either locally or by reacting on the general system, simulate pulmonary phthisis.

2. These lesions are often amenable to treatment; however grave and important they may appear, they are far from being always beyond the resources of art.

3. Consequently, when a case of pulmonary lesion presents itself, it is important, unless the existence of tuberculosis can be certainly made out, to ascertain if the lesion can be traced to syphilis. It is necessary to remember that syphilis is a possible cause of phthisis.

4. When syphilis is suspected of being the cause, the primary indication is to prescribe specific treatment, as such has been known to produce gratifying results.

An important practical contribution to this subject is the report of five cases by Dr. L. McLane Tiffany—the first case, as far as known to the present writer, reported by an American surgeon.

*On Syphilis of the Brain and Nervous System.*—The monograph by Heubner is at once the most comprehensive and the most complete upon the subject in the language. Monographs and clinical reports have also been published by Buzzard, Broadbent, Fournier, Wunderlich, Erlenmayer, Taylor and others. The possibility of syphilis acting as a cause of psychical and nervous disturbances must be borne in mind in obscure cases. The treatment most successful is iodide of potassium in large doses.

In a paper on "Early Syphilis in the Negro," Dr. J. E. Atkinson, gives the results of one hundred cases observed by him in that race. The subject discussed by the author is of great importance to every physician practicing in the South, and is handled by one who is fully conversant with it in all its details. The article is so much condensed that an abstract of it within the limits at our command is impossible.

*The Treatment of Syphilis by means of the Hypodermic Injection* of mercuric bi-chloride, (Lewin) chloro-albuminate and mercuric peptone, (Staub, Bamberger, Grunfeld, Neumann, etc.) and calomel, (Kolliker) has lately been extensively pursued by the authors named, and superiority claimed for each of them. The difficulty of applying this method in private practice, and the disagreeable results—as abscesses and circumscribed dermatitis—which not onfrequently occur, will necessarily limit its application. The veteran syphilidologist, Sigmund, on the other hand, in a recent lecture, reviews the new methods of treatment, and concludes that subcutaneous injection is only suitable in mild cases, and that in severe cases he always relies with more certainty upon the old methods.

Of decided novelty are the views of Keyes upon "The Action of Mercury." By examining the blood with a measuring apparatus known as the *hematimetre*, he found that mercury in small doses, in health or disease, increases the red globules. He has arrived at the following conclusions, among others, as the result of experiment:

"Syphilis diminishes the number of red corpuscles below the healthy standard.

"Mercury, in small doses, continued for a short or a long period in syphilis, alone or with the iodide of potassium, increases the number of red corpuscles in the blood, and maintains a high standard of the same.

"Mercury, in small doses, is a tonic to individuals in fair health—not syphilitic. In such individuals it increases the number of red blood corpuscles."

Drawing these conclusions from his investigations and clinical experience, Keyes was led to adopt what he terms the "tonic treatment" in syphilis. This consists in beginning with very small doses of mercury ($\frac{1}{48}$th gr. corrosive sublimate, $\frac{1}{6}$th gr. proto-iodide, $\frac{1}{2}$ gr. blue mass) three times a day, and increasing the same—carefully watching its effects—until the constitutional effect of the medicine (abdominal pains, diarrhœa, slight stomatitis) are manifested. This is termed "full doses." When this is ascertained, it is continued until the symptoms for which mercury was administered have disappeared, when the dose is dimished one-half—this is the patient's "tonic dose." This is continued steadily from two to two and a half years. Where rapid action is necessary, to obtain the effect of the "full dose," inunction or the mercurial vapor bath may be combined with the internal administration of the remedy. Of course the general condition of the patient as to diet, hygiene, etc., is neglected.

In common with nearly all syphilidologists of the present day, Keyes advocates the use of mercury altogether in the early stages of syphilis; the "mixed treatment"—mercury and iodides—in late secondary eruptions, and the iodides in the gummy lesions wherever occurring. In this stage—tertiary—the author's faith in the iodides is strong, and is manifested in this apotheosis of the potassium salt:

"Under the kindly influence of iodine, unsparingly pushed, the node melts away, the gummy tumor becomes absorbed, the spreading ulcer blushes with healthy granulations, the palsied muscle regains its contractibility, the veil drops from the clouded intellect, the maniacal paroxysm is followed by peace.

"No means in the physicians hands place him so near the Deity as the iodide of potassium. With it, in well-selected syphilitic cases, he can sometimes almost effect a resurrection. Wasted and lost functions are restored; the mind, the memory, the speech, the hearing, the sight, the taste, the touch—all may be recovered by its aid. The prognosis in the most desperate conditions is always infinitely better where syphilis can be made out as having caused the trouble, than where any other diseased condition is at fault; and no amount of destructive tertiary disease need occasion despondency so long as the integrity of the stomach can be preserved, and the physician is strong in his faith in iodine and expert in his methods of using it.

That Dr. Keyes is not a homœopathist in his use of this drug, we may gather from the statement that he has "given *an ounce a day* of the iodide of potassium (ʒij every six hours) in several cases with advantage." He adds, however, "it is very exceptional to give such large doses."

Notwithstanding the clear directions given by Dr. Keyes, and the undoubted superiority of his plan in some cases, it may be doubted whether it will supersede the old-fashioned methods, and especially the inunction cure, combined with the internal use of iron and in the late stages, the iodides.

Bilbache gives a good summary of the history and treatment of syphilis and the so-called chancroid. His recommendations for the prevention of venereal diseases would, if adopted, be a long step on the road of progress, although falling short of what we think is not only desirable, but practicable. We think the attempted mollification of the pietistic elements of society is out of place in a scientific work in our day. Things should be called by their right names. Let us not employ false pretenses. When we mean "the legal and sanitary control of prostitutes," let us say so, and not attempt to hide our meaning under a more ambiguous, though perhaps less offensive, phrase.— *Virginia Medicat Monthly.*

